# Supplementary material for: The interactome of intact mitochondria by cross-linking mass spectrometry provides evidence for coexisting respiratory supercomplexes
Source: Mol Cell Proteomics. 2017 Dec 8;17(2):216–32. doi: 10.1074/mcp.RA117.000470 (PMC5795388; doi:10.1074/mcp.RA117.000470)
Supplement: Supplemental Data [file supp_RA117.000470_133922_0_supp_23978_fzffwf.zip › spectra_annotation/mito_spectra_annotation/255-1-7-1-13-1.pdf]

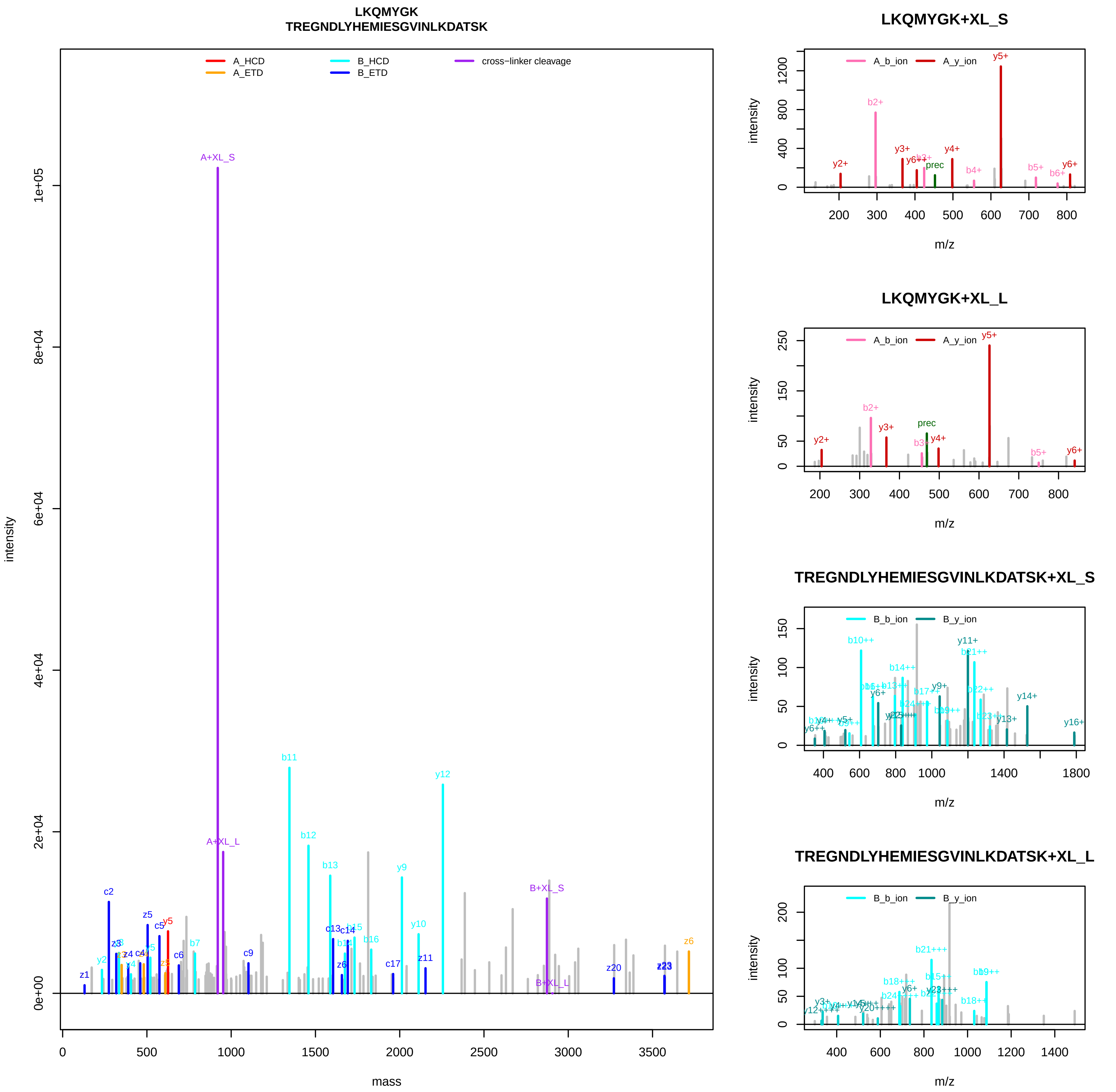

LKQMYGK
TREGNDLYHEMIESGVINLKDATSK
A_HCD
A_ETD
B_HCD
B_ETD
cross−linker cleavage
A+XL_S
A+XL_L
B+XL_S
B+XL_L
b11
b12
b13
b15
b16
b7
y2
y5
y9
y10
y12
c2
c5
c6
c9
c13
c14
c17
z1
z3
z5
z6
z11
z20
z23
intensity
mass
0e+00
2e+04
4e+04
6e+04
8e+04
1e+05
0
500
1000
1500
2000
2500
3000
3500
LKQMYGK+XL_S
A_b_ion
A_y_ion
b2+
b3+
b4+
b5+
b6+
y2+
y3+
y4+
y5+
y6+
y6++
prec
intensity
m/z
LKQMYGK+XL_L
TREGNDLYHEMIESGVINLKDATSK+XL_S
B_b_ion
B_y_ion
b10++
b14++
b17++
b21++
b22++
y6+
y9+
y11+
y13+
y14+
y16+
TREGNDLYHEMIESGVINLKDATSK+XL_L
b18+++
b21+++
b18++
b19++
y6+
y23+++
y20+++
